# Enabling the electrochemical simulation of Li-ion battery electrodes with anisotropic tortuosity in COMSOL Multiphysics^Ⓡ^

**DOI:** 10.1016/j.mex.2021.101425

**Published:** 2021-06-23

**Authors:** Vishwas Goel, Katsuyo Thornton

**Affiliations:** Department of Materials Science and Engineering, University of Michigan, Ann Arbor, MI, United States

**Keywords:** Anisotropic tortuosity, 3D architectures, Comsol Li-ion battery module

## Abstract

Li-ion battery electrodes, such as widely used graphite anodes, may have anisotropic tortuosity due to the non-equiaxed shape of the active material particles and the post-casting calendaring process. Such anisotropy can be ignored in conventional electrodes because all the macroscopic ion transport occurs along the electrode thickness, making the ion transport effectively one dimensional. However, the anisotropy becomes important to consider with three-dimensional architectures, such as those generated by laser-patterning. COMSOL Multiphysics^Ⓡ^ is one of the leading tools used by the Li-ion battery modeling community to simulate the electrochemical dynamics of the Li-ion batteries. However, in its current implementation of the underlying model equations, the Li-ion battery module in COMSOL 5.4 cannot be used to simulate the electrochemical behavior of an electrode with anisotropic tortuosity. In this work, we show how the current implementation can be modified to simulate the anisotropic case.

• The existing Li-ion battery model in COMSOL 5.4 was extended to account for the anisotropy in the electrode tortuosity
• The extended model is necessary to accurately simulate the electrochemical dynamics of 3D architectures such as *Highly ordered laser-patterned electrode (HOLE)*
• The testing and validation results are included in this work

## Graphical abstract

**Figure:**
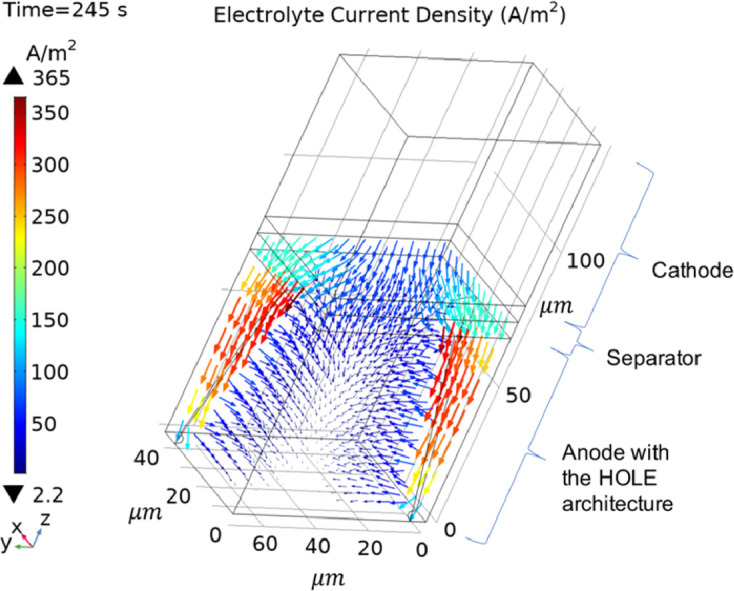

Specifications table

**Table:** 

Subject Area:	Materials Science
More specific subject area:	Electrochemical Systems
Method name:	A method for enabling the electrochemical simulation of Li-ion battery electrodes with anisotropic tortuosity in COMSOL Multiphysics^Ⓡ^
Name and reference of original method:	The Li-ion battery module in COMSOL 5.4
Resource availability:	Not applicable

## Introduction

Li-ion batteries have become immensely popular mobile energy storage devices in our modern lives because of their high energy density and moderately long life. To gain further insights into the underlying mechanisms and to further improve the performance of these devices, electrochemical simulations are widely used. These simulations are based on the model developed by Doyle at al. [1], [2], [3] and its variants, which are built on the porous electrode theory (PET) [4,5].

Standard Li-ion battery electrodes exhibit anisotropic tortuosity in the electrolyte phase, owing to the non-equiaxed shape of the active material particles and the manufacturing technique, post-casting calendaring, used to make them. For example, a graphite anode was reported to have a through-plane (along the electrode thickness) tortuosity factor that is four times the in-plane value, for an electrode porosity of 40% [6]. However, models typically do not need to account for the difference in tortuosity because the macroscopic ion-transport occurs only along the thickness in conventional electrodes if the edge effects [7] are insignificant. Thus, it is sufficient to set tortuosity, (or alternatively the effective diffusivity), to correspond to that for the through-thickness direction. On the other hand, when advanced, three-dimensional electrode architectures like highly ordered laser-patterned electrodes (HOLE) [8] are considered, it becomes necessary to account for the anisotropic tortuosity in the electrochemical simulations. In such architectures, the gradients in the electrolyte concentration and potential are three dimensional, and thus provide driving force for transport in all three directions.

COMSOL Multiphysics^Ⓡ^ is a widely used tool by the Li-ion battery modeling community for solving the coupled partial differential equations of the Doyle model [3] in conventional electrodes [9], [10], [11]. Despite COMSOL's capability to solve the model equations in three dimensions, the current implementation of the model equations (specifically in COMSOL 5.4) cannot be directly applied to simulate the electrochemical performance of electrodes with anisotropic tortuosity. To overcome this limitation, we propose a modification to the COMSOL software. Furthermore, we used the modified implementation to simulate the performance of HOLE graphite anodes with and without anisotropic tortuosity. Our results highlight the importance of considering the effect of anisotropic tortuosity in such electrodes.

## Method details

The existing implementation of the model equations in COMSOL can be accessed from the porous electrode sub-node in the Li-ion battery module by enabling the “Equation View” option. Table 1 summarizes all the input values required by COMSOL for solving the equations pertaining to the ion-transport inside the electrolyte phase of a porous electrode. The other model equations can be found here [8].

**Table 1:** List of inputs that are required by COMSOL for simulating the ion-transport within the electrolyte phase of a porous electrode.

Variable	Units	Definition
F	C/mol	Faraday's constant
R	J/(mol K)	Universal gas constant
T	K	Temperature of the simulation
$\in_{l}$	–	Volume fraction of the electrolyte phase
$\sigma_{l}=\left[\begin{array}{ccc} \sigma_{l,xx} & \sigma_{l,xy} & \sigma_{l,xz}\\ \sigma_{l,yx} & \sigma_{l,yy} & \sigma_{l,yz}\\ \sigma_{l,zx} & \sigma_{l,zy} & \sigma_{l,zz} \end{array}\right]$	S/m	Intrinsic electrolyte conductivity (tensor)
$D_{l}$	m²/s	Intrinsic electrolyte diffusivity (scalar)
$t_{+}$	–	Transference number for Li-ions
$\frac{\partial \text{ln}(f_{\pm })}{\partial \text{ln}(c_{l})}$	–	Salt activity dependence on the salt concentration
$f(\in_{l})$	–	Ratio between the effective and intrinsic electrolyte properties (diffusivity and conductivity)

It can be noted from Table 1 that COMSOL allows for an ionic conductivity tensor $\sigma_{l}$ to be input into the model, while only a scalar input value is allowed for the ionic diffusivity $D_{l}$. Since the ionic conductivity and diffusivity are related in both the diluted and concentrated binary electrolytes [12], this disparate treatment for these two quantities is unphysical. Therefore, one needs to be cautious while inputting these quantities into the model if applying the model beyond the intended 1D/pseudo-1D cases.

Table 2 summarizes the implementation of all the ion-transport equations in a porous medium in COMSOL. The quantities $c_{l}$ and $\phi_{l}$ represent the electrolyte concentration (mol/m^3^) and the electrolyte potential (V), respectively. The reaction source term is denoted by $R_{l}$, which is equal to $i_{total}/F$. The quantity $i_{total}$ represents the total reaction current per unit volume of the porous media.

**Table 2:** Implementation of the ion-transport equations in COMSOL.

Eq. No.	Equation	Units	Description
3.	$i_{x}=i_{lx}+\frac{2RT\left(1+\frac{\partial ln(f_{\pm })}{\partial ln(c_{l})}\right)\left(1-t_{+}\right)f\left(\in_{l}\right)\left(\sigma_{l,xx}\frac{\partial c_{l}}{\partial x}\;+\;\sigma_{l,xy}\frac{\partial c_{l}}{\partial y}\;+\;\sigma_{l,xz}\frac{\partial c_{l}}{\partial z}\right)}{F(max(c_{l},0.1))}$	A/m²	X component of the electrolyte current density
4.	$i_{y}=i_{ly}+\frac{2RT\left(1+\frac{\partial ln(f_{\pm })}{\partial ln(c_{l})}\right)\left(1-t_{+}\right)f\left(\in_{l}\right)\left(\sigma_{l,yx}\frac{\partial c_{l}}{\partial x}\;+\;\sigma_{l,yy}\frac{\partial c_{l}}{\partial y}\;+\;\sigma_{l,yz}\frac{\partial c_{l}}{\partial z}\right)}{F(max(c_{l},0.1))}$	A/m²	Y component of the electrolyte current density
5.	$i_{z}=i_{lz}+\frac{2RT\left(1+\frac{\partial ln(f_{\pm })}{\partial ln(c_{l})}\right)\left(1-t_{+}\right)f\left(\in_{l}\right)\left(\sigma_{l,zx}\frac{\partial c_{l}}{\partial x}\;+\;\sigma_{l,zy}\frac{\partial c_{l}}{\partial y}\;+\;\sigma_{l,zz}\frac{\partial c_{l}}{\partial z}\right)}{F(max(c_{l},0.1))}$	A/m²	Z component of the electrolyte current density
6.	$i_{lx}=-f\left(\in_{l}\right)\left(\sigma_{l,xx}\frac{\partial \phi_{l}}{\partial x}\;+\;\sigma_{l,xy}\frac{\partial \phi_{l}}{\partial y}+\;\sigma_{l,xz}\frac{\partial \phi_{l}}{\partial z}\right)$	A/m²	X component of the electrolyte current density due to migration
7.	$i_{ly}=-f\left(\in_{l}\right)\left(\sigma_{l,yx}\frac{\partial \phi_{l}}{\partial x}\;+\;\sigma_{l,yy}\frac{\partial \phi_{l}}{\partial y}+\;\sigma_{l,yz}\frac{\partial \phi_{l}}{\partial z}\right)$	A/m²	Y component of the electrolyte current density due to migration
8.	$i_{lz}=-f\left(\in_{l}\right)\left(\sigma_{l,zx}\frac{\partial \phi_{l}}{\partial x}\;+\;\sigma_{l,zy}\frac{\partial \phi_{l}}{\partial y}+\;\sigma_{l,zz}\frac{\partial \phi_{l}}{\partial z}\right)$	A/m²	Z component of the electrolyte current density due to migration
9.	$N_{Li^{+},x}=-f\left(\in_{l}\right)D_{l}\frac{\partial c_{l}}{\partial x}+\frac{i_{x}t_{+}}{F}$	mol/(m²s)	X component of the Li-ion flux in the electrolyte
10.	$N_{Li^{+},y}=-f\left(\in_{l}\right)D_{l}\frac{\partial c_{l}}{\partial y}+\frac{i_{y}t_{+}}{F}$	mol/(m²s)	Y component of the Li-ion flux in the electrolyte
11.	$N_{Li^{+},z}=-f\left(\in_{l}\right)D_{l}\frac{\partial c_{l}}{\partial z}+\frac{i_{z}t_{+}}{F}$	mol/(m²s)	Z component of the Li-ion flux in the electrolyte
12.	$N_{PF_{6}^{-},x}=-f\left(\in_{l}\right)D_{l}\frac{\partial c_{l}}{\partial x}-\frac{i_{x}\left(1-t_{+}\right)}{F}$	mol/(m²s)	X component of the anion (${\text{PF}}_{6}^{-}$) flux in the electrolyte
13.	$N_{PF_{6}^{-},y}=-f\left(\in_{l}\right)D_{l}\frac{\partial c_{l}}{\partial y}-\frac{i_{y}\left(1-t_{+}\right)}{F}$	mol/(m²s)	Y component of the anion (${\text{PF}}_{6}^{-}$) flux in the electrolyte
14.	$N_{PF_{6}^{-},z}=-f\left(\in_{l}\right)D_{l}\frac{\partial c_{l}}{\partial z}-\frac{i_{z}\left(1-t_{+}\right)}{F}$	mol/(m²s)	Z component of the anion (${\text{PF}}_{6}^{-}$) flux in the electrolyte
15.	$\in_{l}\frac{\partial c_{l}}{\partial t}=\;f\left(\in_{l}\right)\left(\frac{\partial }{\partial x}D_{l}\frac{\partial c_{l}}{\partial x}\;+\frac{\partial }{\partial y}D_{l}\frac{\partial c_{l}}{\partial y}+\frac{\partial }{\partial z}D_{l}\frac{\partial c_{l}}{\partial z}\right)+R_{l}-\frac{i_{total}t_{+}}{F}-\frac{\frac{\partial t_{+}}{\partial x}.i_{x}+\frac{\partial t_{+}}{\partial y}.i_{y}+\frac{\partial t_{+}}{\partial z}.i_{z}}{F}$	mol/(m^3^s)	Mass conservation equation in the electrolyte

For a porous medium, the effective transport property of the medium is a function of the intrinsic value of the property, the phase volume fraction, and the tortuosity [13]. For instance, the effective electrode diffusivity $D_{eff}$ in a porous electrode is obtained as [13]

(1) $$D_{eff}=\frac{\in_{l}}{\tau^{2}}D_{l},$$

where τ represents the electrode tortuosity. The effective electrolyte conductivity is calculated in the same manner by substituting the corresponding value of intrinsic conductivity in Eq. (1). Furthermore, we refer to the ratio of the effective transport property to the corresponding intrinsic value as the correction factor,

(2) $$f\left(\in_{l}\right)=\frac{D_{eff}}{D_{l}}=\frac{\in_{l}}{\tau^{2}}.$$

Thus, it can be seen that the correction factor is a function of the electrode tortuosity. Now for an electrode with anisotropic effective diffusivity (due to an anisotropy in the microstructure), the correction factor would have different values along different axes. As mentioned before, the current COMSOL implementation assumes the electrode to have isotropic tortuosity, and thus, it only uses one correction factor $f(\in_{l})$ to calculate the current densities and ion fluxes in all three directions (Eq. 3–14), and for defining the mass conservation equation (Eq. 15). To account for the effect of the anisotropic tortuosity, we modified the COMSOL implementation by replacing the single correction factor $f(\in_{l})$ in all the equations by the direction-specific correction factor $f_{j}\left(\in_{l}\right)$, where j=x,y,or z. The modified equations are listed in Table 3 along with a comparison with the existing equations. It should be noted that while doing so, one needs to select “No correction” in the “Effective Transport Parameter Correction” sub-menu in the porous electrode sub-node, and then modify the ion-transport equations, as discussed above. The correction factors, $f_{x}\left(\in_{l}\right)$, $f_{y}\left(\in_{l}\right)$, and $f_{z}\left(\in_{l}\right)$ should be treated as parameters, and their value should be set in the parameter table under “Global Definitions.”

**Table 3:** Side by side comparison of the existing implementation of the ion-transport equations in COMSOL, and the modified implementation to simulate the anisotropic tortuosity case.

Implementation in COMSOL	Modified Implementation
$i_{x}=i_{lx}+\frac{2RT\left(1+\frac{\partial ln(f_{\pm })}{\partial ln(c_{l})}\right)\left(1-t_{+}\right)f\left(\in_{l}\right)\left(\sigma_{l,xx}\frac{\partial c_{l}}{\partial x}\;+\;\sigma_{l,xy}\frac{\partial c_{l}}{\partial y}\;+\;\sigma_{l,xz}\frac{\partial c_{l}}{\partial z}\right)}{F(max(c_{l},0.1))}$	$i_{x}=i_{lx}+\frac{2RT\left(1+\frac{\partial ln(f_{\pm })}{\partial ln(c_{l})}\right)\left(1-t_{+}\right)f_{x}\left(\in_{l}\right)\left(\sigma_{l,xx}\frac{\partial c_{l}}{\partial x}\right)}{F(max(c_{l},0.1))}$
$i_{y}=i_{ly}+\frac{2RT\left(1+\frac{\partial ln(f_{\pm })}{\partial ln(c_{l})}\right)\left(1-t_{+}\right)f\left(\in_{l}\right)\left(\sigma_{l,yx}\frac{\partial c_{l}}{\partial x}\;+\;\sigma_{l,yy}\frac{\partial c_{l}}{\partial y}\;+\;\sigma_{l,yz}\frac{\partial c_{l}}{\partial z}\right)}{F(max(c_{l},0.1))}$	$i_{y}=i_{\mathrm{ly}}+\frac{2\mathrm{RT}\left(1+\frac{\partial \ln(f_{\pm })}{\partial \ln(c_{l})}\right)\left(1-t_{+}\right)f_{y}\left(\in_{l}\right)\left(\sigma_{l,\mathrm{yy}}\frac{\partial c_{l}}{\partial y}\right)}{F(\max\left(c_{l},0.1\right))}$
$i_{z}=i_{lz}+\frac{2RT\left(1+\frac{\partial ln(f_{\pm })}{\partial ln(c_{l})}\right)\left(1-t_{+}\right)f\left(\in_{l}\right)\left(\sigma_{l,zx}\frac{\partial c_{l}}{\partial x}\;+\;\sigma_{l,zy}\frac{\partial c_{l}}{\partial y}\;+\;\sigma_{l,zz}\frac{\partial c_{l}}{\partial z}\right)}{F(max(c_{l},0.1))}$	$i_{z}=i_{lz}+\frac{2RT\left(1+\frac{\partial ln(f_{\pm })}{\partial ln(c_{l})}\right)\left(1-t_{+}\right)f_{z}\left(\in_{l}\right)\left(\sigma_{l,zz}\frac{\partial c_{l}}{\partial z}\right)}{F(max(c_{l},0.1))}$
$i_{lx}=-f\left(\in_{l}\right)\left(\sigma_{l,xx}\frac{\partial \phi_{l}}{\partial x}\;+\;\sigma_{l,xy}\frac{\partial \phi_{l}}{\partial y}+\;\sigma_{l,xz}\frac{\partial \phi_{l}}{\partial z}\right)$	$i_{\mathrm{lx}}=-f_{x}\left(\in_{l}\right)\left(\sigma_{l,\mathrm{xx}}\frac{\partial \phi_{l}}{\partial x}\right)$
$i_{ly}=-f\left(\in_{l}\right)\left(\sigma_{l,yx}\frac{\partial \phi_{l}}{\partial x}\;+\;\sigma_{l,yy}\frac{\partial \phi_{l}}{\partial y}+\;\sigma_{l,yz}\frac{\partial \phi_{l}}{\partial z}\right)$	$i_{ly}=-f_{y}\left(\in_{l}\right)\left(\sigma_{l,yy}\frac{\partial \phi_{l}}{\partial y}\right)$
$i_{lz}=-f\left(\in_{l}\right)\left(\sigma_{l,zx}\frac{\partial \phi_{l}}{\partial x}\;+\;\sigma_{l,zy}\frac{\partial \phi_{l}}{\partial y}+\;\sigma_{l,zz}\frac{\partial \phi_{l}}{\partial z}\right)$	$i_{lz}=-f_{z}\left(\in_{l}\right)\left(\sigma_{l,zz}\frac{\partial \phi_{l}}{\partial z}\right)$
$N_{Li^{+},x}=-f\left(\in_{l}\right)D_{l}\frac{\partial c_{l}}{\partial x}+\frac{i_{x}t_{+}}{F}$	$N_{Li^{+},x}=-f_{x}\left(\in_{l}\right)D_{l}\frac{\partial c_{l}}{\partial x}+\frac{i_{x}t_{+}}{F}$
$N_{Li^{+},y}=-f\left(\in_{l}\right)D_{l}\frac{\partial c_{l}}{\partial y}+\frac{i_{y}t_{+}}{F}$	$N_{Li^{+},y}=-f_{y}\left(\in_{l}\right)D_{l}\frac{\partial c_{l}}{\partial y}+\frac{i_{y}t_{+}}{F}$
$N_{Li^{+},z}=-f\left(\in_{l}\right)D_{l}\frac{\partial c_{l}}{\partial z}+\frac{i_{z}t_{+}}{F}$	$N_{Li^{+},z}=-f_{z}\left(\in_{l}\right)D_{l}\frac{\partial c_{l}}{\partial z}+\frac{i_{z}t_{+}}{F}$
$N_{PF_{6}^{-},x}=-f\left(\in_{l}\right)D_{l}\frac{\partial c_{l}}{\partial x}-\frac{i_{x}\left(1-t_{+}\right)}{F}$	$N_{PF_{6}^{-},x}=-f_{x}\left(\in_{l}\right)\frac{\partial c_{l}}{\partial x}-\frac{i_{x}\left(1-t_{+}\right)}{F}$
$N_{PF_{6}^{-},y}=-f\left(\in_{l}\right)D_{l}\frac{\partial c_{l}}{\partial y}-\frac{i_{y}\left(1-t_{+}\right)}{F}$	$N_{PF_{6}^{-},y}=-f_{y}\left(\in_{l}\right)D_{l}\frac{\partial c_{l}}{\partial y}-\frac{i_{y}\left(1-t_{+}\right)}{F}$
$N_{PF_{6}^{-},z}=-f\left(\in_{l}\right)D_{l}\frac{\partial c_{l}}{\partial z}-\frac{i_{z}\left(1-t_{+}\right)}{F}$	$N_{PF_{6}^{-},z}=-f_{z}\left(\in_{l}\right)D_{l}\frac{\partial c_{l}}{\partial z}-\frac{i_{z}\left(1-t_{+}\right)}{F}$
$\in_{l}\frac{\partial c_{l}}{\partial t}=\;f\left(\in_{l}\right)\left(\frac{\partial }{\partial x}D_{l}\frac{\partial c_{l}}{\partial x}\;+\frac{\partial }{\partial y}D_{l}\frac{\partial c_{l}}{\partial y}+\frac{\partial }{\partial z}D_{l}\frac{\partial c_{l}}{\partial z}\right)+R_{l}-\frac{i_{total}t_{+}}{F}-\frac{\frac{\partial t_{+}}{\partial x}.i_{x}+\frac{\partial t_{+}}{\partial y}.i_{y}+\frac{\partial t_{+}}{\partial z}.i_{z}}{F}$	$\in_{l}\frac{\partial c_{l}}{\partial t}=\;\left(f_{x}\left(\in_{l}\right)\frac{\partial }{\partial x}D_{l}\frac{\partial c_{l}}{\partial x}\;+f_{y}\left(\in_{l}\right)\frac{\partial }{\partial y}D_{l}\frac{\partial c_{l}}{\partial y}+f_{z}\left(\in_{l}\right)\frac{\partial }{\partial z}D_{l}\frac{\partial c_{l}}{\partial z}\right)+R_{l}-\frac{i_{total}t_{+}}{F}-\frac{\frac{\partial t_{+}}{\partial x}.i_{x}+\frac{\partial t_{+}}{\partial y}.i_{y}+\frac{\partial t_{+}}{\partial z}.i_{z}}{F}$

We note that the suggested modification assumes that the off-diagonal components in the ionic conductivity tensor (Entry 5, Table 1) are zero. Further modifications would be needed when the off-diagonal components are non-zero. Since it is assumed that the ionic conductivity tensor is diagonal, we removed all the off-diagonal terms from the current density equations (Eq. 3–8, Table 2), as shown in Table 3. Finally, we note that although the variables that represent the ion-fluxes (Eq. 9–14, Table 2) are not used in the mass conservation equation (Eq. 15, Table 2), their modification is required as the values for the ion-fluxes are stored for post-simulation analyses.

The equations for the electronic current density $i_{s}$ can be modified in a similar manner, as shown in Table 4. The potential of the active material phase in the porous electrode is denoted by $\phi_{s}$. The isotropic correction factor is denoted by $g(\in_{s})$, where $\in_{s}$ is the volume fraction of the active material in a porous electrode. The anisotropic correction factors are denoted by $g_{j}\left(\in_{s}\right)$, where j=x,y,or z. The electronic conductivity of the active material is denoted by $\sigma_{s}$, which is treated as a tensor in COMSOL's current implementation. Therefore, as with ionic conductivity, the earlier discussion above about the applicability of the suggested modification for a diagonal tensor also holds here. It should be noted that no other model equation was modified in this work. We call the model with the modified equations as the modified battery model from hereon.

**Table 4:** Side by side comparison of the existing implementation of the electronic current density equations in COMSOL and the modified implementation to simulate the anisotropic tortuosity case.

Implementation in COMSOL	Modified Implementation
$i_{s,x}=-\;g\left(\in_{s}\right)\left(\sigma_{s,xx}\frac{\partial \phi_{s}}{\partial x}\;+\sigma_{s,xy}\frac{\partial \phi_{s}}{\partial y}+\sigma_{s,xz}\frac{\partial \phi_{s}}{\partial z}\right)$	$i_{s,x}=-g_{x}\left(\in_{s}\right)\left(\sigma_{s,\mathrm{xx}}\frac{\partial \phi_{s}}{\partial x}\right)$
$i_{s,y}=-\;g\left(\in_{s}\right)\left(\sigma_{s,yx}\frac{\partial \phi_{s}}{\partial x}\;+\sigma_{s,yy}\frac{\partial \phi_{s}}{\partial y}+\sigma_{s,yz}\frac{\partial \phi_{s}}{\partial z}\right)$	$i_{s,y}=-g_{y}\left(\in_{s}\right)\left(\sigma_{s,yy}\frac{\partial \phi_{s}}{\partial y}\right)$
$i_{s,z}=-\;g\left(\in_{s}\right)\left(\sigma_{s,zx}\frac{\partial \phi_{s}}{\partial x}\;+\sigma_{s,zy}\frac{\partial \phi_{s}}{\partial y}+\sigma_{s,zz}\frac{\partial \phi_{s}}{\partial z}\right)$	$i_{s,z}=-g_{z}\left(\in_{s}\right)\left(\sigma_{s,zz}\frac{\partial \phi_{s}}{\partial z}\right)$

## Testing and verification

Our proposed modification in the model equations affects only the diffusion and migration dynamics of ions within a porous electrode. Therefore, the modification can be tested and verified by comparing individually the diffusion and migration dynamics obtained from the modified model with the solutions of the diffusion and migration equations, respectively, as described below.

Two tests were performed to examine our proposed modification, namely, the diffusion dynamics test and the migration dynamics test. Since our modification is valid for any porous electrode, we carried out the proposed modification only in the anode for the purpose of these tests. In each test, we first simulated the anisotropic dynamics, under certain boundary conditions, using the modified battery model.

Thereafter, we compared the spatial distribution of the relevant physical quantity (electrolyte concentration for the diffusion dynamics test and electrolyte current density for the migration dynamics test) with those obtained from numerical solutions of the diffusion/migration equation in a three-dimensional domain with the same dimensions as the anode and subjected to the same boundary conditions.

**Fig. 1:**
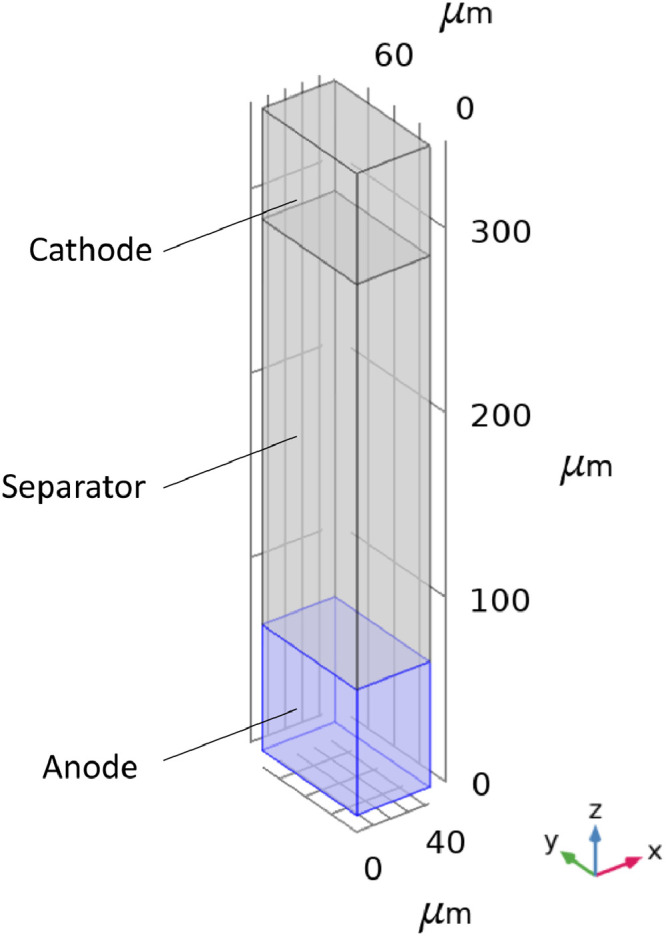
Schematic of the cell geometry used in the modified battery model. The highlighted region represents the anode.

The model geometry used in the modified battery model is shown in Fig. 1. The geometry consists of three domains: a cathode on the top, an anode at the bottom, and a separator in between. The model parameters employed in the tests are listed in Table 5. For more details on the model, the reader is referred to our previous publication [8].

**Table 5:** List of quantities used in the validation along with their values.

Quantity	Units	Value
Cell Length (along Y-axis)	μm	73.61
Cell Width (along X-axis)	μm	42.50
Cell Thickness (along Z-axis)	μm	328
Anode Thickness ($L_{Z}$, along Z-axis)	μm	68
$f_{x}\left(\in_{l}\right)$ (anode)	–	0.0458
$f_{y}\left(\in_{l}\right)$ (anode)	–	0.0458
$f_{z}\left(\in_{l}\right)$ (anode)	–	0.0208
$D_{l}$	$m^{2}/s$	4.04× 10^−10^
$\sigma_{l}$	S/m	1

### Diffusion dynamics test

For the diffusion dynamics test, we compared the evolution of the electrolyte concentration obtained from the modified battery model under a specific set of boundary conditions with that obtained from the numerical solution of the diffusion equation in a three-dimensional domain with a corresponding setup.

**Fig. 2:**
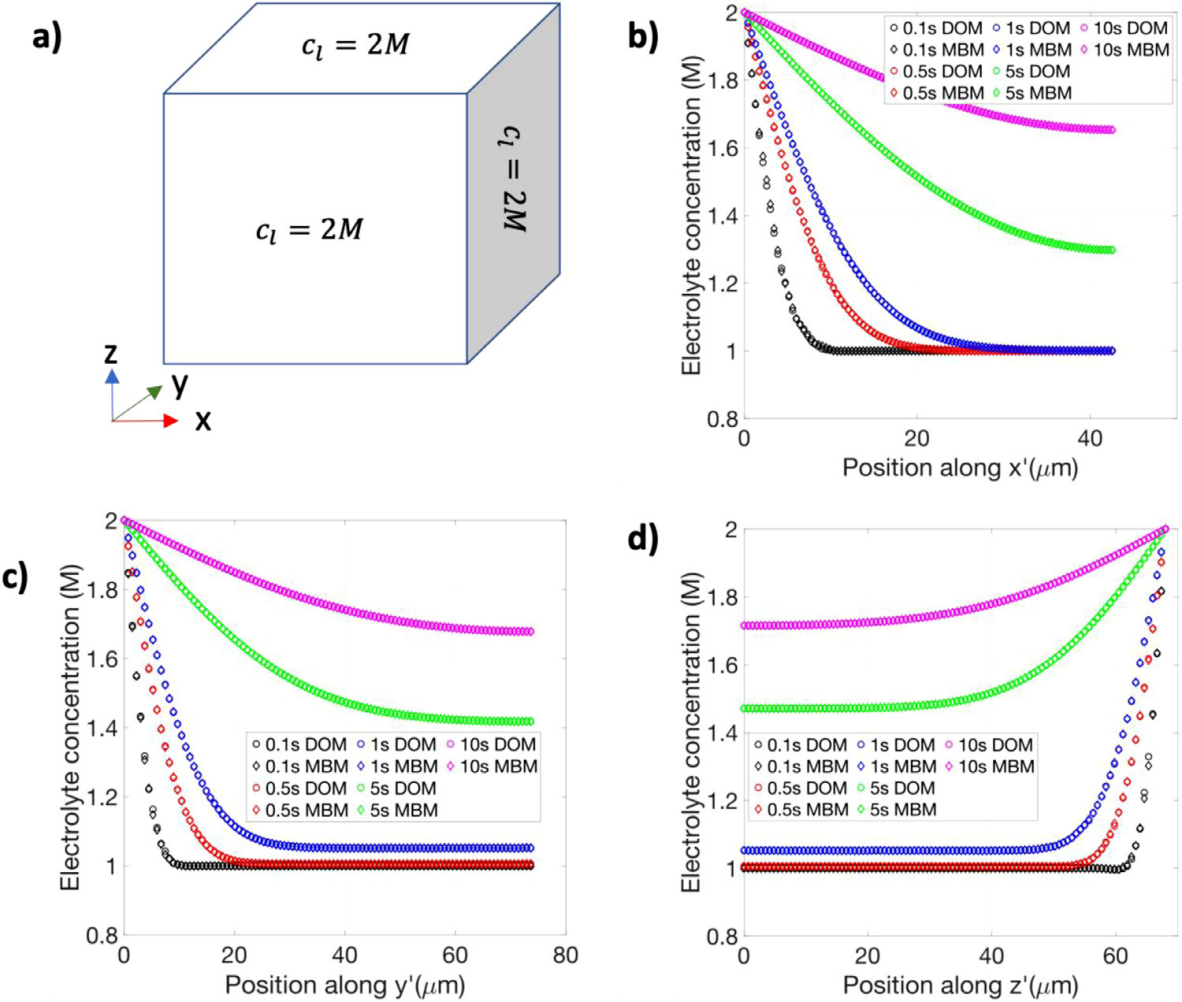
a Schematic of the boundary conditions applied in the anode region of the three-dimensional cell geometry. b-d Comparison of the concentration evolution obtained from the modified battery model (MBM) and the diffusion-only model (DOM) along x’, y’, and z’, respectively, which have origin at the centroid of the anode.

The conditions for the anisotropic diffusion test are as follows. The initial electrolyte concentration in the cell was set at 1 M. Dirichlet boundary conditions ($c_{l}\;=\;2M$) were imposed at three of the anode faces, as shown in Fig. 2a. No-flux boundary conditions were imposed at all other external faces of the domain, and zero-current boundary condition was imposed at the topmost face. The last boundary condition ensures the absence of any migration effect. The model was then used to simulate the electrolyte concentration evolution.

The comparison data was generated by numerically solving the diffusion equation in COMSOL. For this purpose, a separate model with only diffusion physics was set up in COMSOL. The diffusion-only model had the same model geometry and boundary conditions as that of the anode in the modified battery model. It is interesting to note that COMSOL supports anisotropic diffusion in the diffusion models, but not in the Li-ion battery module. The diffusion model was also used to simulate the concentration evolution. The two sets of results for the concentration evolution were then compared along three axes x’, y’, and z’, which are parallel to the x, y, and z axes, respectively, and have their origin at the centroid of the anode geometry. The comparison of the results along each axis is shown in Figs. 2b-d for few select times. Qualitatively, the results from the two models match well at all the shown times for all the axes. We also evaluated the error quantitatively by examining the infinity norm of the difference between the concentration values obtained by the line cuts along x’, y’, and z’ at each time shown in Fig. 2b-d. The maximum error (among different times) was found to be 0.019 M, 0.018 M, and 0.029 M for x’, y’, and z’, respectively. In other words, the maximum error between the two datasets is less than 3% for a reference concentration value of 1 M. The excellent qualitative and quantitative match demonstrates that the modified battery model is correctly solving the anisotropic diffusion dynamics, and hence, verifies our proposed modification.

### Migration dynamics test

In a similar fashion as the diffusion dynamics test, we compared the two sets of the spatial distribution of the electrolyte current densities along each axis obtained from the modified battery model and a migration-only model under certain conditions. The migration-only model was set up using the “secondary current distribution” module in COMSOL with the anode geometry.

The conditions for the test are as follows. The initial electrolyte concentration in the cell was set at 1 M and the transference number for the Li-ions in the anode was set equal to 1. These two conditions ensure that the electrolyte concentration remains constant throughout the anode. Additionally, the electrochemical reaction in the anode was set to be zero, and no external current was applied to the cell. These conditions ensure that only ionic migration, and not diffusion, contributes to the ionic current.

**Fig. 3:**
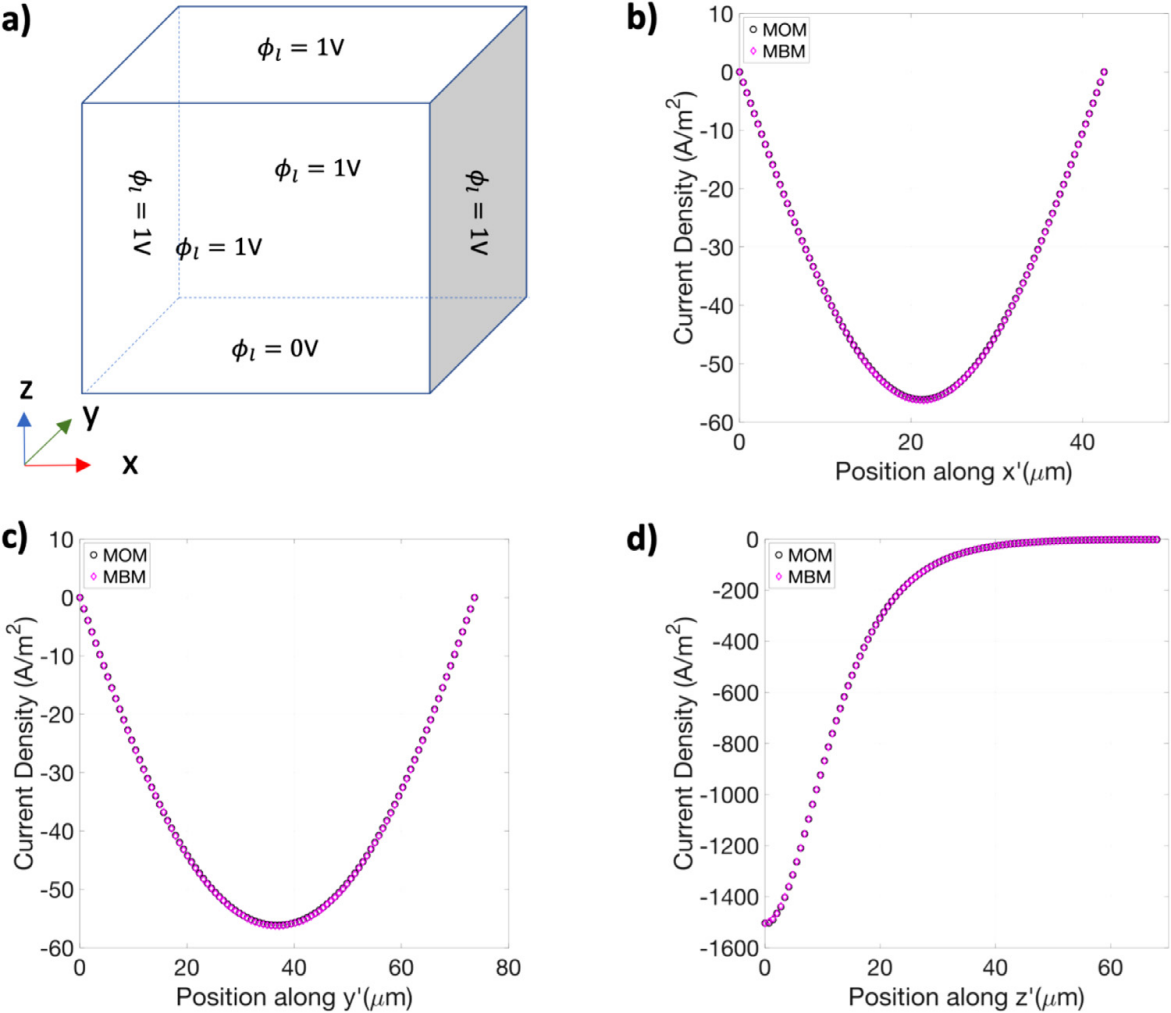
a Schematic of the boundary conditions applied in the anode region of the three-dimensional cell geometry. b-d Comparison of the z component of the current density vector obtained from the modified battery model (denoted by MBM) and the migration-only model (denoted by MOM) along x’, y’, and z’, respectively, which have origin at the centroid of the anode.

The boundary conditions were set as follows. Dirichlet boundary conditions were applied to all the faces of the anode. Specifically, the electrolyte potential $\phi_{l}$ was set to be 0 V at the bottom face of the anode, and 1 V at all other faces of the anode, respectively, as shown in Fig. 3a. The migration-only model has the same material properties and boundary conditions as the anode in the modified battery model. Fig. 3b-d compare the z component of the steady-state current density vector obtained from the two models along x’, y’, and z’. The steady-state value is chosen for the comparison because the system achieves steady-state instantly. It can be seen that the qualitative match between the results from the two models is excellent. To quantitatively compare the results, the error was defined in the same manner as the diffusion dynamic test, i.e., as the infinity norm of the difference between the two sets of results on three line cuts passing through the centroid of the anode region. The error was found to be 5.1 A/m^2^, 0.2 A/m^2^, and 0.18 A/m^2^, for the x’, y’, and z’ axes, respectively, which corresponds to less than 1% error for the typical current density values shown in Fig. 3b-d. Thus, the proposed implementation accurately solved the anisotropic migration dynamics.

## Effect of anisotropic tortuosity

We used the modified model to simulate the effect of anisotropic tortuosity on the dynamics during the 4C charging of a typical graphite anode with ~3 mAh/cm^2^ loading and HOLE architecture [8]. In particular, we considered anodes with and without anisotropic tortuosity. For the isotropic case, $f_{x}\left(\in_{l}\right)\;=\;f_{y}\left(\in_{l}\right)\;=\;f_{z}\left(\in_{l}\right)\;=\;0.0208$. Whereas for the anisotropic case, $f_{z}\left(\in_{l}\right)\;=\;0.0208$ and $f_{x}\left(\in_{l}\right)\;=\;f_{y}\left(\in_{l}\right)\;=\;2.2f_{z}\left(\in_{l}\right)\;=0.0458$. The anisotropy factor of 2.2 was obtained from Ref. [8]. It should be noted that the thickness of the anode was aligned along z axis and the value of $f_{z}\left(\in_{l}\right)$ was kept the same in both the cases. The HOLE pattern used in the simulations had cylindrical channels with a hexagonal symmetry and a channel length equal to the anode thickness. The inter-channel spacing and the channel diameter were set to 75 μm and 25 μm, respectively. All other model details can be found in our previous work [8].

**Fig. 4:**
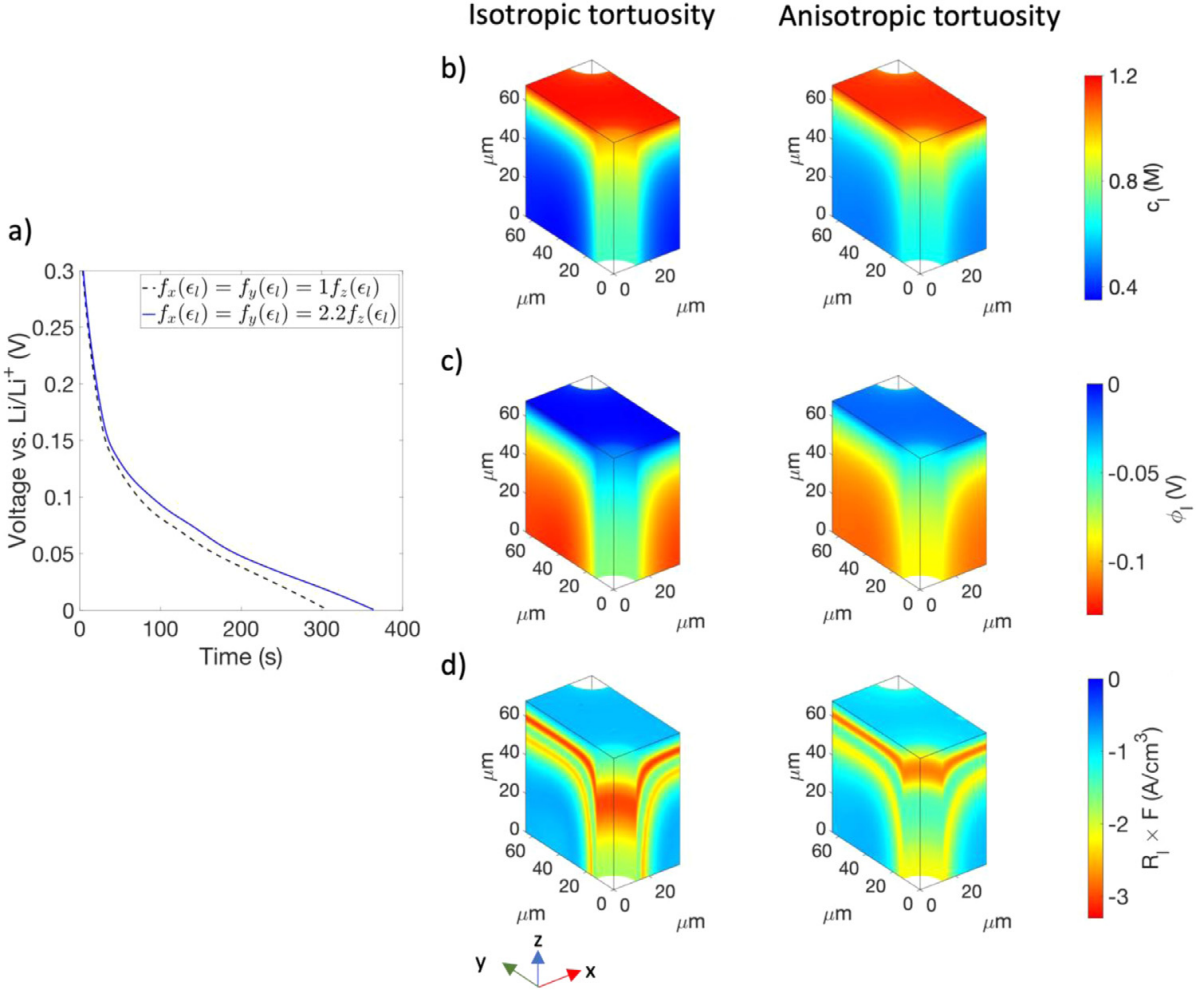
a Comparison of the simulated voltage vs. time plots for anodes with isotropic tortuosity (black dashed curve) and anisotropic tortuosity (blue solid curve). Comparison of the distribution of (b) $c_{l}$, (c) $\phi_{l}$, and (d) $R_{l}\times F$ (reaction current density per unit volume of the anode) for the two anodes at t=306 s. The top and bottom surface of anodes in this figure represent the anode/separator and anode/current collector interfaces, respectively.

The simulation results are summarized in Fig. 4. Fig. 4a shows the comparison of the voltage vs. time plot for the two cases. As can be seen, the anode with anisotropic tortuosity sustains 4C charging for a longer duration before reaching 0 V than the one with isotropic tortuosity (364 s vs. 306 s). The simulations were terminated at 0 V because below it Li plating might occur and the model does not include the corresponding physics [8]. The enhanced performance is a result of more facile transport of Li-ions along x and y axes in the electrolyte, which is set by the higher values of $f_{x}\left(\in_{l}\right)$ and $f_{y}\left(\in_{l}\right)$. Furthermore, this facile transport in the in-plane directions indirectly facilitates the transport along z axis.

To demonstrate this effect, we compare the spatial distribution of the electrolyte concentration within the anode for the isotropic (left column) and anisotropic (right column) cases in Fig. 4b at t=306 s, which corresponds to the time when the anode with isotropic tortuosity reaches 0 V (and at which there is a risk for lithium metal plating). It can be seen that the in-plane concentration gradients in the region within a distance of 40 μm from the anode/current collector interface (bottom surface of the anode) are much smaller in the anode with anisotropic tortuosity than the one with isotropic tortuosity. Moreover, it can be noted that the gradient along z axis is much smaller for the anisotropic case than the isotropic case even though both anodes have the same value of $f_{z}\left(\in_{l}\right)$. A similar trend can be observed in the electrolyte potential, which is shown in Fig. 4c at t=306 s. Finally, with more facile transport of Li-ions in the anode with anisotropic tortuosity, the distribution of the reaction current density becomes more homogeneous (Fig. 4d), which results in the longer duration of sustained 4C charging for the anode [14]. Thus, it can be seen that ignoring the effect of anisotropic tortuosity can lead to significant underestimation (~16% in this case) of the charge stored in the electrode during a fast-charging simulation.

In conclusion, the results discussed in this section highlight the importance of considering the effect of anisotropic tortuosity while studying the electrochemical performance of electrodes with three-dimensional architectures.

## Conclusion

In this work, we have shown how the existing implementation of the Doyle model in COMSOL 5.4 can be enhanced to account for anisotropic tortuosity in porous electrodes. We described the implementation details of our proposed modification and provided verification results that were compared to numerical solutions. Furthermore, we demonstrated the application of the modified implementation to simulate the 4C performance of a laser patterned (HOLE architecture) graphite anode with and without anisotropic tortuosity.

## Declaration of Competing Interests

The authors declare that they have no known competing financial interests or personal relationships that could have appeared to influence the work reported in this paper.
